# Optimized production and immunogenicity of an insect virus-based chikungunya virus candidate vaccine in cell culture and animal models

**DOI:** 10.1080/22221751.2021.1886598

**Published:** 2021-02-25

**Authors:** Awadalkareem Adam, Huanle Luo, Samantha R. Osman, Binbin Wang, Christopher M. Roundy, Albert J. Auguste, Kenneth S. Plante, Bi-Hung Peng, Saravanan Thangamani, Elena I. Frolova, Ilya Frolov, Scott C. Weaver, Tian Wang

**Affiliations:** a Department of Microbiology & Immunology, University of Texas Medical Branch, Galveston, TX, USA; b Department of Entomology, Fralin Life Science Institute, Virginia Polytechnic Institute and State University, Blacksburg, VA, USA; c Center for Emerging, Zoonotic, and Arthropod-borne Pathogens, Virginia Tech, Blacksburg, VA, USA; d World Reference Center for Emerging Viruses and Arboviruses, Institute for Human Infections and Immunity, University of Texas Medical Branch, Galveston, TX, USA; e Department of Neuroscience, Cell Biology and Anatomy, University of Texas Medical Branch, Galveston, TX, USA; f Department of Microbiology and Immunology, SUNY Upstate Medical University, Syracuse, NY, USA; g Department of Microbiology, University of Alabama at Birmingham, Birmingham, AL, USA; h Sealy Institute for Vaccine Sciences, University of Texas Medical Branch, Galveston, TX, USA; i Department of Pathology, University of Texas Medical Branch, Galveston, TX, USA

**Keywords:** Vaccine, insect, immunity, Chikungunya virus, safety

## Abstract

A chimeric Eilat/ Chikungunya virus (EILV/CHIKV) was previously reported to replicate only in mosquito cells but capable of inducing robust adaptive immunity in animals. Here, we initially selected C7/10 cells to optimize the production of the chimeric virus. A two-step procedure produced highly purified virus stocks, which was shown to not cause hypersensitive reactions in a mouse sensitization study. We further optimized the dose and characterized the kinetics of EILV/CHIKV-induced immunity. A single dose of 10^8^ PFU was sufficient for induction of high levels of CHIKV-specific IgM and IgG antibodies, memory B cell and CD8^+^ T cell responses. Compared to the live-attenuated CHIKV vaccine 181/25, EILV/CHIKV induced similar levels of CHIKV-specific memory B cells, but higher CD8^+^ T cell responses at day 28. It also induced stronger CD8^+^, but lower CD4^+^ T cell responses than another live-attenuated CHIKV strain (CHIKV/IRES) at day 55 post-vaccination. Lastly, the purified EILV/CHIKV triggered antiviral cytokine responses and activation of antigen presenting cell (APC)s *in vivo,* but did not induce APCs alone upon *in vitro* exposure. Overall, our results demonstrate that the EILV/CHIKV vaccine candidate is safe, inexpensive to produce and a potent inducer of both innate and adaptive immunity in mice.

## Introduction

Chikungunya virus (CHIKV) belongs to the *Togaviridae* family of alphaviruses. It is transmitted by *Aedes* mosquitoes and causes chikungunya fever, which is often accompanied by severe, debilitating and chronic arthralgia [1]. The virus was initially associated with human disease in the 1950s in Tanzania and has re-emerged over the last decade to cause epidemics in Africa, Asia, Europe, and the Americas [2,3]. Human vaccines are currently not available for CHIKV infection. Multiple strategies have been used for CHIKV vaccine development, including formalin-inactivated CHIKV [4], virus-like particles (VLP) [5,6], a live measles virus-vectored version [7,8], a live-attenuated vaccine in late phase clinical trials [9], the live-attenuated CHIKV 181/25 strain [10,11] and its nsP2 mutant [12], CHIKV/IRES [13,14], and chimeric alphavirus-based vaccines [15-17], which have been shown to be effective and exhibit immunogenicity. However, the possibility of evolution of live recombinant viruses to more pathogenic phenotypes, inefficient and expensive production of VLPs and inactivated vaccines remain as concerns. Therefore, in this study, we further developed a fundamentally new strategy of vaccine development based on using Eilat alphavirus (EILV)-CHIKV chimera, whose replication machinery is functional only in mosquito cells.

EILV is a mosquito-specific alphavirus. It replicates well in mosquito cells but is not capable of replication in vertebrate cells. Its host restriction in vertebrate cells is known to occur at both entry and, and the RNA replication steps [18]. EILV/CHIKV, a chimeric virus was recently constructed to contain the structural proteins of CHIKV (E1, 6 K, E2, E3 and capsid) and the non-structural (NS) proteins (nsP1, nsP2, nsP3 and nsP4) of EILV. The chimeric virus remains capable of replication in mosquito cells, but not in vertebrate cells [19]. Furthermore, vaccination with a single dose of EILV/CHIKV in mice and non-human primates (NHPs) induces CHIKV-specific adaptive immune responses and protects host from wild-type CHIKV challenge. This suggests that EILV/CHIKV can be further developed as a safe and efficacious CHIKV vaccine candidate [20]. Here, we initially characterized and optimized the production and purification of EILV/CHIKV in mosquito cells. Next, we optimized the immune responses of purified EILV/CHIKV in immunocompetent C57BL/6 (B6) mice and characterized the kinetics of EILV/CHIKV-induced immunity. A single minimal dose of 10^8^ PFU EILV/CHIKV induced a strong protective antibody response, and memory B cell (MBC) and CD8^+^ T cell responses. Compared to the live-attenuated CHIKV181/25 and CHIKV/IRES, EILV/CHIKV triggered similar levels of MBCs but stronger and long-lasting CD8^+^ T cell responses. It also quickly boosted antiviral cytokine production and induced the activation of antigen presenting cells (APC)s in mice, but did not activate APCs alone upon *in vitro* exposure. Our results demonstrate that the purified chimeric virus is a potent inducer of innate and adaptive immunity.

## Methods

*Cells*: C7/10 cells were obtained from Henry Huang (Washington University, St. Louis, MO). C6/36 cells were obtained from the American Type Culture Collection (ATCC, Manassas, VA). Both cell lines were propagated at 28°C and 5% CO_2_ in Dulbecco’s modified Eagle’s medium (DMEM) supplemented with 10% heat-inactivated (HI) foetal bovine serum (FBS) and 10% tryptase phosphate broth (TPB).

*Virus growth and purification*: C7/10 cells were seeded at 2 × 10^7^ cells per 150-mm dish 24 h prior the infection. They were infected with 2 × 10^6^ PFU of EILV/CHIKV in phosphate-buffered saline (PBS), supplemented with 1% FBS for 1 h at 28°C. Cells were washed with PBS and further incubated for 18 h in VP-SF media (Invitrogen) supplemented with 10% TPB and glutamine. HEPES buffer pH 7.5 was added to the harvested virus-containing media. Next, media was filtered through 0.22 μm filter and passed at room temperature through Cellufine sulfate (AMSBIO) column equilibrated with PBS. After loading the entire volume of the media, column was washed with PBS, and virus was eluted in a minimal volume (1–1.5 ml) of 7 x PBS. It was immediately loaded on the top of freshly prepared sucrose gradient (1.5 ml of 50%, 2 ml of 40% and 7 ml of 30% prepared on PBS) and centrifuged at 36,000 rpm, 4°C for 3.5 h. A well visible band was collected, diluted with PBS, and aliquots were stored at −80°C. Viral titers were evaluated in the harvested media and in the samples collected after each step of purification. Presence of mosquito proteins in virion preparations has been analysed by mass spectrometry. Equal amounts of virions purified on Cellufine sulfate column or after additional purification by sucrose gradient were loaded on SDS-PAGE. The gel was run for ∼1.5 cm, stained with Coomassie and gel segments containing proteins were excised. The in-gel digestion with trypsin and mass spectrometry analysis have been performed in UAB Mass Spectrometry/Proteomics Shared Facility. Data were searched against custom database containing Aedes albopictus and viral proteins. Final data were filtered in Scaffold Viewer with minimum protein threshold set at 99%, peptide threshold at 50% and minimum number of peptide was set at 2.

*Plaque assay*: C7/10 cells were seeded at concentration of 1.5 x10^6^ cells per well. After incubation for 6-8 h at 28°C, they were infected with serial 10-fold dilutions of viral samples. After incubation for 1 h at 28°C, the inocula were replaced with DMEM, supplemented with 0.6% tragacanthin gum, 5% HI FBS and 5% TPB. After 2–2.5 days of incubation at 28°C, cells were fixed in 2.5% formaldehyde for at least 10 min and stained with Crystal violet. Plaque assays of vertebrate-competent control vaccines and challenge virus were performed on Vero cells at 37°C using standard methods [21].

*Mice*: Four to six-week-old B6 mice were bred and maintained at the University of Texas Medical Branch (UTMB) Animal facility. Mice were inoculated intraperitoneally (i.p.) with 10^4^–10^8^ PFU EILV/CHIKV, 10^5^ PFU CHIKV/IRES, 5.5 × 10^5^ PFU CHIKV 181/25, or formalin-inactivated CHIKV. All animal experiments were approved by the Animal Care and Use Committee at UTMB.

*Mice sensitization study*: 4-6-week old B6 mice were sensitized with 50 females *Ae. albopictus* mosquitoes for 3 times on a 14-day interval. On day 43, mice were injected i.d. on footpad with 10 µg *Ae. albopictus* salivary gland extract (SGE), 10^8^ PFU of EILV/CHIKV, or PBS (mock). 24 h after challenge, mice were euthanized to collect tissues. Footpad was removed and fixed in 4% PFA for H&E staining. Spleens were also isolated and treated with SGE *in vitro* with 20 μg/ml and supernatant were collected to measure cytokines.

*SGE preparation*: *Ae. albopictus* from established laboratory colonies maintained at the University of Texas Medical Branch, were used in these studies. Mosquitoes were reared in an insectary, maintained at 28°C with 80% relative humidity and a 14-h light/10-h dark photoperiod. Adults were allowed to feed on 10% sucrose diet *ad libitum*. At 5–9 days post-eclosion, female mosquitoes were cold anesthetized, and salivary glands were removed, under a dissection microscope, from the mosquito body. Two hundred pairs of female salivary glands were collected into sterile water and macerated to generate SGE. The final volume of the SGE was adjusted to 200 μl so each μl of SGE corresponds to one salivary gland pair. The mosquito SGE were stored at −80°C.

*Quantitative PCR (Q-PCR)*: Viral-infected cells or tissues were re-suspended in Trizol (Invitrogen) for RNA extraction. Complementary (c) DNA was synthesized by using a qScript cDNA synthesis kit (Bio-Rad, Hercules, CA). The sequences of the primer sets for cytokines and PCR reaction conditions were described previously [22,23]. The PCR assay was performed in the CFX96 real-time PCR system (Bio-Rad). Gene expression was calculated using the formula 2^ ^-[C^t^(target gene)-C^t^(*β-actin*)]^ as described before [24].

*Flow cytometry*: Splenocytes were stained with antibodies for CD11c, CD80, CD86, F4/80 (e-Biosciences, San Diego, CA). After staining, the cells were fixed with 1% paraformaldehyde in PBS and examined using a C6 flow cytometer (BD Biosciences). Dead cells were excluded on the basis of forward and side light scatter. Data were analysed with a CFlow Plus flow cytometer (BD Biosciences).

*Intracellular cytokine staining (ICS)*: Fresh isolated or frozen splenocytes from vaccinated mice or controls were stimulated with 50 ng/ml PMA and 500 ng/ml ionomycin (Sigma-Aldrich) for 4 h or CD8^+^ T cell-restricted CHIKV E1 peptide (HSMTNAVTI) [25,26] for 5 h at 37°C. Golgi-plug (BD Biosciences) was added at the beginning of stimulation. Cells were harvested, stained with Abs for CD4 or CD8, fixed in 2% paraformaldehyde and permeabilized with 0.5% saponin before adding PE-conjugated anti-IFN-γ, or control PE-conjugated rat IgG1. Samples were processed with a C6 Flow Cytometer instrument. Dead cells were excluded on the basis of forward and side scatter. Data were analysed with a CFlow Plus Flow Cytometer (BD Biosciences).

*ELISA*: Sera from vaccinated mice and controls were collected at indicated time points. The plates were coated with EILV/CHIKV (5 x10^4^ PFU per well) overnight at 4°C as described previously [27], washed twice with PBS, containing 0.05% Tween-20 (PBS-T) and blocked with 8% FBS for 2.5 h. Sera diluted 1:40 in blocking buffer was added for 1 h followed by incubation with goat anti-mouse IgG or IgM (Sigma-Aldrich) coupled to alkaline phosphatase (1:1000 dilution) for 1 h. Colour was developed with *p*-nitrophenyl phosphate (Sigma-Aldrich) and the intensity was read at an absorbance of 405 nm.

*ELISPOT assay*: The assays were performed as previously described [28] with some modifications. Briefly, splenocytes were stimulated with 1 µg/ml R848 and 10 ng/ml recombinant human IL-2 (Mabtech In, OH). Millipore ELISPOT plates (Millipore Ltd, Darmstadt, Germany) were coated with EILV/CHIKV (1 × 10^8^ PFU/ well), CHIKV VLP (The Native Antigen Company, Oxford, UK) 15 μg/ml, or anti-human Ig capture Ab (Mabtech In). Cells were harvested and added in duplicates to assess CHIKV-specific or total IgG antibody-secreting cells (ASC)s. The plates were incubated overnight at 37°C, followed by biotin conjugated anti-mouse IgG (Mabtech In) for 2 h at room temperature, and then streptavidin-ALP for 1 h. Plates were developed with BCIP/NBT-Plus substrate until distinct spots emerge, washed and scanned using an ImmunoSpot4.0 analyzer and the spots were counted with ImmunoSpot software (Cellular Technology Ltd, Cleveland, OH) to determine the spot-forming cells (SFC) per 10^6^ splenocytes.

*Cytokine Bioplex*: Sera were harvested, and cytokine production was measured by using a Bio-Plex Pro Mouse Cytokine Assay (Bio-Rad).

*Statistical analysis*: Values for viral load, cytokine production, and antibody and T cell responses experiments were compared using Prism software (GraphPad) statistical analysis and were presented as means ± SEM. *P* values of these experiments were calculated with a non-paired Student’s *t* test.

## Results

### Production and purification of EILV/CHIKV in mosquito cells

**Figure 1.**
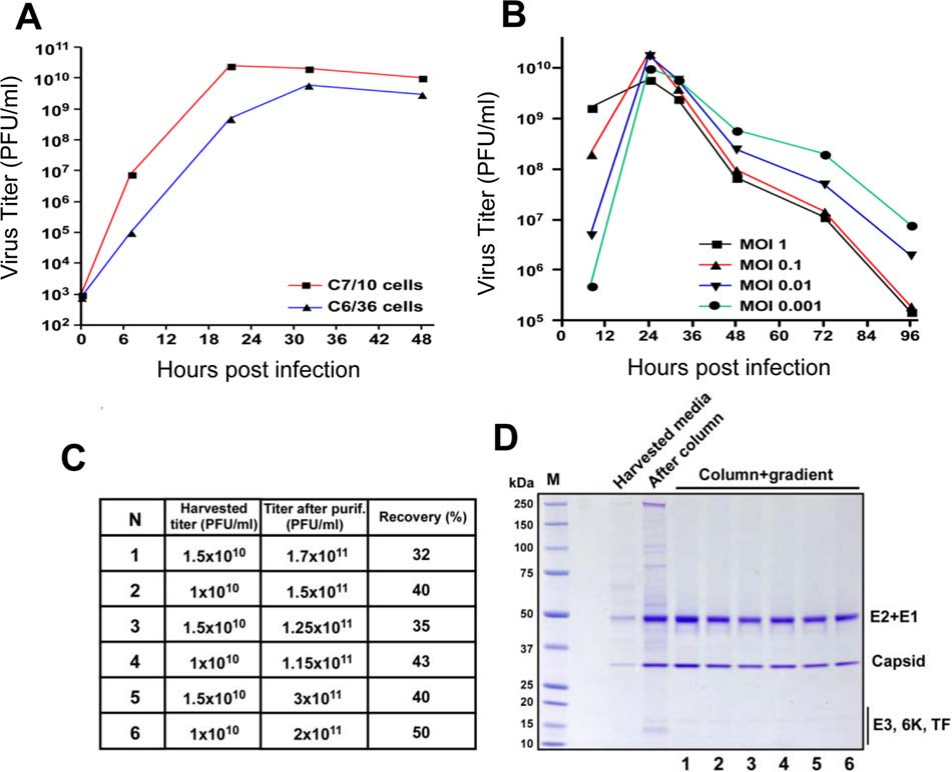
Production and purification of EILV/CHIKV in mosquito cells. (A) C6/36 cells and C7/10 cells were infected with EILV/CHIKV (MOI 0.05) and then incubated at 28°C. At the indicated time points, media were replaced, and titers were determined by plaque assay on C7/10 cells. This experiment was reproducibly repeated 2 times. The results of one of them are presented. (B) C7/10 cells were infected with EILV/CHIKV at different MOIs. Media were harvested at the indicated time-points, and infectious titers were determined by plaque assay on C7/10 cells. (C,D). EILV/CHIKV samples were produced and purified as described in Materials and Methods. Samples were collected from different stages to determine the viral titers and purity. C. Comparative analysis of 6 different batches of purified viruses at different stages of purification. (D) Viral presence was detected by Coomassie staining in the harvested media.

**Figure 2.**
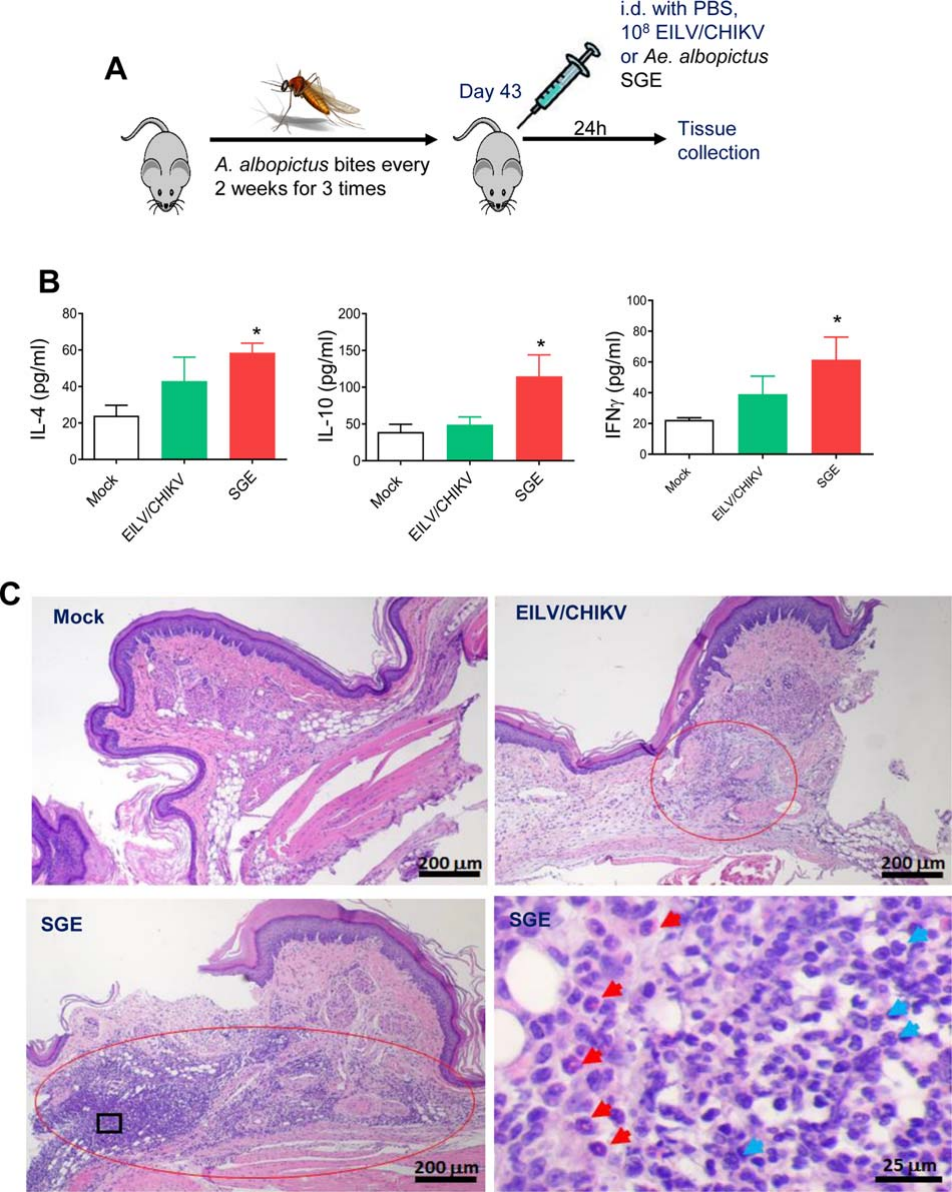
Hypersensitivity study in mice. B6 mice were sensitized by exposure to female *Ae. albopictus* mosquitoes three times during a 14-day period. On day 43, mice were injected i.d. on footpad with 10 µg SGE protein, 10^8^ PFU of EILV/CHIKV or PBS (mock). 24 h after challenge, mouse tissues were harvested to assess allergy responses. (A) Scheme of sensitization study. *(*B) Splenocytes were stimulated with 20 μg/ml SGE for 48 h and culture supernatants were collected to measure cytokine production by Bioplex. n = 5. * *P<* 0.05 compared to mock group. (C) H &E staining of footpad inflammation. Mock: no inflammation. EILV/CHIKV: minor inflammation in the subcutaneous tissue with mononuclear cells, eosinophils and few neutrophils. SGE: right panel shown enlarged image of the black rectangular box marked in the left panel. Severe inflammation in the subcutaneous tissue with large amounts of eosinophils (red arrows) and neutrophils (blue arrows) as well as mononuclear cells.

One important goal to develop EILV/CHIKV as a vaccine candidate is to design an inexpensive and efficient methodology for production and purification of the chimeric virus. We initially applied two lines of *Aedes albopictus* cells to compare their ability to support EILV/CHIKV replication. As previously described for other alphaviruses [29], C7/10 cells supported EILV/CHIKV replication at higher rates and to higher titers than the C6/36 cells (Figure 1(A)). Thus, C7/10 cells were selected for further development of production and purification protocol. They were infected at different multiplicities of infection (MOI)s and infectious titers of the released into the culture medium virus were determined at different times post infection (pi, Figure 1(B)). These experiments were aimed at identifying the optimal MOI and time of harvesting. Cell cultures infected at MOIs of 0.01–0.1 demonstrated the highest titers. At an MOI of 0.1, by 8 h pi, viral titers were sufficient for infecting all of the cells, and by 24 h pi, titers were higher than 10^10^ plaque-forming unit (PFU)/ ml. Based on these characteristics, C7/10 cells were infected at an MOI of 0.05 for 8 h. Then the incubation continued for 18 h in serum-free VP-SF medium. The released virus was harvested, and purified and concentrated by a two-step purification method, which started with chromatography on Cellufine sulfate column followed by ultracentrifugation in discontinuous sucrose gradients (see Methods for details). This simple purification procedure was fast and highly reproducible in removal of mosquito proteins. Examples of titers and virion purity in several independent purification are presented in Figure 1(C,D). Viral presence was detectable by Coomassie staining in the harvested media, and column purification was clearly insufficient for generating homogeneous samples. After ultracentrifugation all the samples demonstrated the presence of CHIKV E2, E1 and capsid proteins. We also analysed the presence of host proteins by mass spectrometry in samples collected on the different stages of purification (Table S1). Large number of mosquito proteins remained present in column-purified/concentrated virions. After additional purification by sucrose gradient only 3 mosquito proteins were detected in virions. Interestingly, these proteins were previously identified in Sindbis virions and are likely integral components of alphavirus particles [30]. NSAF values suggest that they likely present as 1–2 molecules per virion. To confirm low mosquito proteins in the viral stocks, we next sensitized mice by exposure to 10–20 *Ae. albopictus* bites every 2 weeks, for a total of 3 exposures, and were challenged with either 10^8^ PFU of purified EILV/CHIKV, with PBS as a negative control (mock), or with an *Ae. albopictus* SGE as a positive control*.* While the SGE group showed induction of Th1 and Th2 cytokines and substantial infiltration of eosinophils (red arrows), neutrophils (blue arrows) and mononuclear cells, neither the EILV/CHIKV nor the negative control groups showed induction of Th1 or Th2 cytokines or more than minimal inflammation in the inoculation sites (Figure 2). Overall, the two-step purification procedure produced high purity of viral stocks.

### A minimal single dose of 10^8^ PFU EILV/CHIKV is required to induce strong CHIKV-specific antibody production, and memory B cell (MBC) responses in mice

**Figure 3.**
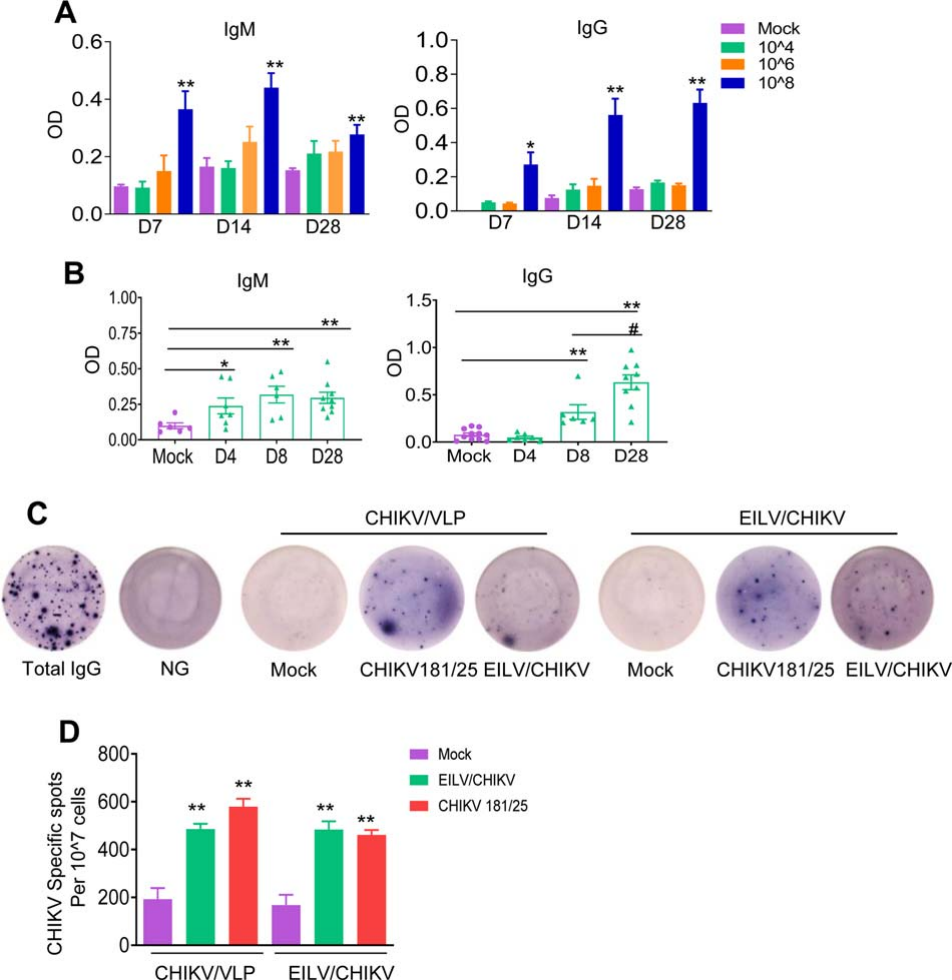
A minimal dose of 10^8^ PFU EILV/CHIKV is required to induce strong CHIKV-specific IgM and IgG production, and memory B cell responses in mice. (A) 4-week-old B6 mice were vaccinated with various doses of EILV/CHIKV or PBS (mock). CHIKV- specific IgM and IgG antibodies in sera were detected by ELISA. n = 4–5. (B) 4-week-old B6 mice were vaccinated with 10^8^ PFU EILV/CHIKV or PBS (mock). At different time post-vaccination, CHIKV- specific IgM and IgG antibodies in sera were detected by ELISA. n = 6–9. C-D. 4-week-old B6 mice were vaccinated with 10^8^ PFU EILV/CHIKV, 5.5 x10^5^ PFU CHIKV 181/25, or PBS (mock). At day 28, CHIKV- specific MBC responses were determined by ELISPOT analysis. (C) Images of wells from MBC culture. (D) Frequencies of CHIKV antibody secreting cells per 10^7^ input cells in MBC cultures from the subject. n = 5–8. ** *P* < 0.01 compared to mock group.

To optimize the immunogenicity of the EILV/CHIKV, serial 100-fold dilutions of EILV/CHIKV ranging from 10^4^ to 10^8^ PFU were tested in 4-to-6-week old B6 mice. Mock vaccinated mice served as controls. At days 7, 14 and 28 post vaccination, blood samples were collected to determine levels of CHIKV-specific IgG and IgM production by ELISA. As shown in Figure 3(A), neither 10^4^ nor 10^6^ PFU of EILV/CHIKV induced CHIKV- specific IgM and IgG production to detectable levels at days 7, 14 and 28 post vaccination. In comparison to the mock group, 10^8^ PFU EILV/CHIKV-vaccinated mice showed enhanced levels of IgM and IgG production at all three time points. We next assessed the kinetics of CHIKV-specific IgG and IgM production in mice vaccinated with 10^8^ PFU EILV/CHIKV. Compared to the mock group, IgM production was increased at days 4 and 8 and remained high at day 28 post-vaccination (Figure 3(B)). A CHIKV-specific IgG response was not detectable until day 8 and continued to increase day 28. The development of MBCs is critical for control of virus infection and dissemination and has been known to be one of the important biomarkers for vaccine efficacy [31]. We utilized conventional B-cell ELISpot to measure CHIKV-specific MBCs in splenocytes of vaccinated mice or controls. Because circulating MBCs do not actively secrete Abs, we stimulated the cells with the TLR7/8 agonist, R848, and rIL-2 *in vitro* for 7 days to convert MBCs into ASCs. Ig capture antibody, EILV/CHIKV, and CHIKV VLP were used as antigens to detect total ASCs, EILV/CHIKV-specific, and CHIKV VLP-specific MBCs respectively. At day 28, we detected CHIKV-specific ASCs in the EILV/CHIKV vaccinated mice. The frequency of CHIKV-specific MBCs was significantly higher in EILV/CHIKV-vaccinated mice than in the mock group, but was similar to that in mice vaccinated with CHIKV 181/25, a live attenuated CHIKV vaccine candidate (Figure 3(C,D)). Overall, these results suggest that a single minimal dose of 10^8^ PFU EILV/CHIKV induced strong antibody production and MBC responses.

### EILV/CHIKV induces long-lasting CD8^+^ T cell responses in mice

**Figure 4.**
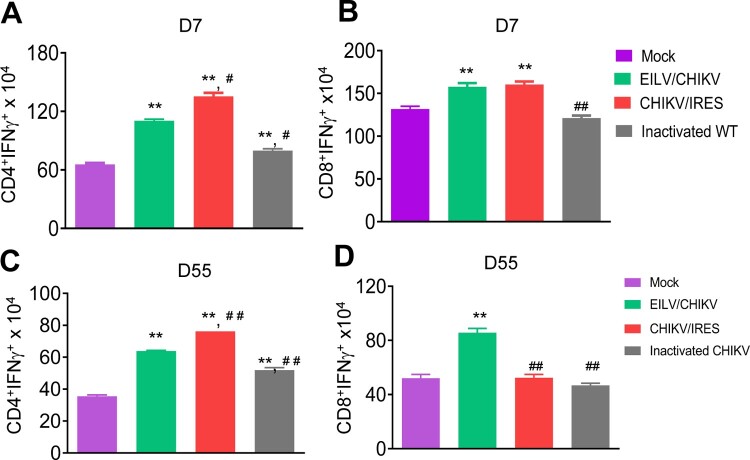
EILV/CHIKV triggers long-lasting CD8^+^ T cell responses compared to CHIKV/IRES in mice. 4-week-old B6 mice were vaccinated with 10^8^ PFU non-purified EILV/CHIKV, 5log_10_ PFU CHIKV/IRES, 8.3 log_10_ PFU formalin-inactivated wild-type CHIKV, or PBS (mock). At day 7 (A,B) and day 55 (C,D) post-vaccination, splenocytes were cultured *ex vivo* with PMA and ionomycin for 4 h, and stained for IFN-γ, CD3, and CD4 or CD8. Total T cells were gated. Total number of IFN-γ^+^ T cell subsets per spleen is shown. n* *= 5–7. **P < 0.01 compared to mock group. ^#^*P* < 0.01 or ^##^*P* < 0.01 compared to EILV/CHIKV vaccinated group. One experiment was shown here.

**Figure 5.**
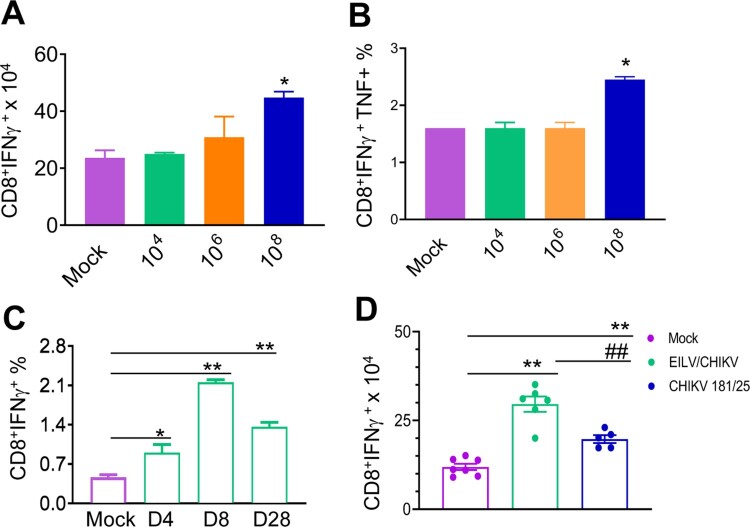
A single dose of 10^8^ PFU of purified EILV/CHIKV elicits strong CD8^+^ T cell responses in mice. 4-week-old B6 mice were vaccinated with EILV/CHIKV, CHIKV 181/25 or PBS (mock). At indicated time points post vaccination, splenocytes were harvested cultured *ex vivo* with CHIKV specific peptide for 5 h, and stained for IFN-γ, TNF-α, CD3, and CD8. Total T cells were gated. (A,B) Day 28 post vaccination. Total number of IFN-γ^+^ (A) and IFNγ^+^TNFα^+^ (B) T cell subsets per spleen is shown. C.Day (D) 4, 8 and 28 post vaccination. Total T cells were gated. Percentage of IFN-γ^+^ T cell subsets is shown. *n *= 2–4. D. Total number of IFN-γ^+^ T cell subsets per spleen at day 28 post vaccination is shown. *n *= 5–7. **P < 0.01, *P < 0.05 compared to mock group. ^##^*P* < 0.01 compared to EILV/CHIKV vaccinated group.

CHIKV/IRES, another live attenuated CHIKV vaccine strain – is known to be safe, effective and induce strong T cell-mediated immune responses in mice [13,14]. Here, we compared the cellular immune responses of mice-vaccinated with 1x 10^8^ PFU cell-culture derived EILV/CHIKV versus 10^5^ PFU CHIKV/IRES CHIKV/IRES. PBS and formalin inactivated CHIKV vaccinated mice were used as controls. At day 7, EILV/CHIKV- and CHIKV/IRES-vaccinated mice showed the induction of CD4^+^ and CD8^+^ T cell responses compared to the mock group (Figure 4(A,B)). In one experiment, we found that the formalin-inactivated CHIKV-vaccinated group had higher CD4^+^ T cell responses than the mock group, but it was much lower compared to the other vaccinated groups. At day 55, while all three groups, including EILV/CHIKV, CHIKV/IRES and formalin inactivated CHIKV vaccinated mice, had stronger CD4^+^ T cell responses than the mock group, only EILV/CHIKV -vaccinated mice demonstrated high levels of CD8^+^ T cell responses (Figure 4(C,D)). Thus, EILV/CHIKV induces long-lasting CD8^+^ T cell responses. To further characterize cellular immunity induced by purified EILV/CHIKV, serial 100-fold dilutions of EILV/CHIKV ranging from 10^4^ to 10^8^ PFU were tested in 4–6-week-old B6 mice. Splenocytes of vaccinated mice were re-stimulated *in vitro* with a CHIKV-specific peptide targeting CD8^+^ T cell epitope, and CHIKV-specific T cell responses were analysed using ICS assay. At day 28, mice vaccinated with 10^8^ PFU of EILV/CHIKV displayed markedly increased numbers and percentages of CHIKV-specific CD8^+^IFN-γ^+^ and CD8^+^IFNγ ^+^ TNFα^+^ T cells, respectively, compared to those of the mock group (Figure 5(A,B)). However, this induction of T cells was not observed in the lower doses vaccinated groups. When examining the kinetics of EILV/CHIKV-mediated CD8^+^ T cell responses, we noted that 10^8^ PFU of purified EILV/CHIKV triggered CHIKV-specific CD8^+^ T cell responses at days 4 and 8 and remained increased at 4-weeks post vaccination (Figure 5(C)). Compared to mice- vaccinated with CHIKV 181/25, the EILV/CHIKV-vaccinated group demonstrated increased numbers of CD8^+^IFN-γ^+^ T cells at 4 weeks post vaccination (Figure 5(D)). Overall, these results suggest that EILV/CHIKV induces rapid and long-lasting CHIKV- specific CD8^+^ T cell responses.

### EILV/CHIKV triggers anti-viral cytokine responses and activation of APCs *in vivo,* but does not induce APCs upon *in vitro* exposure

**Figure 6.**
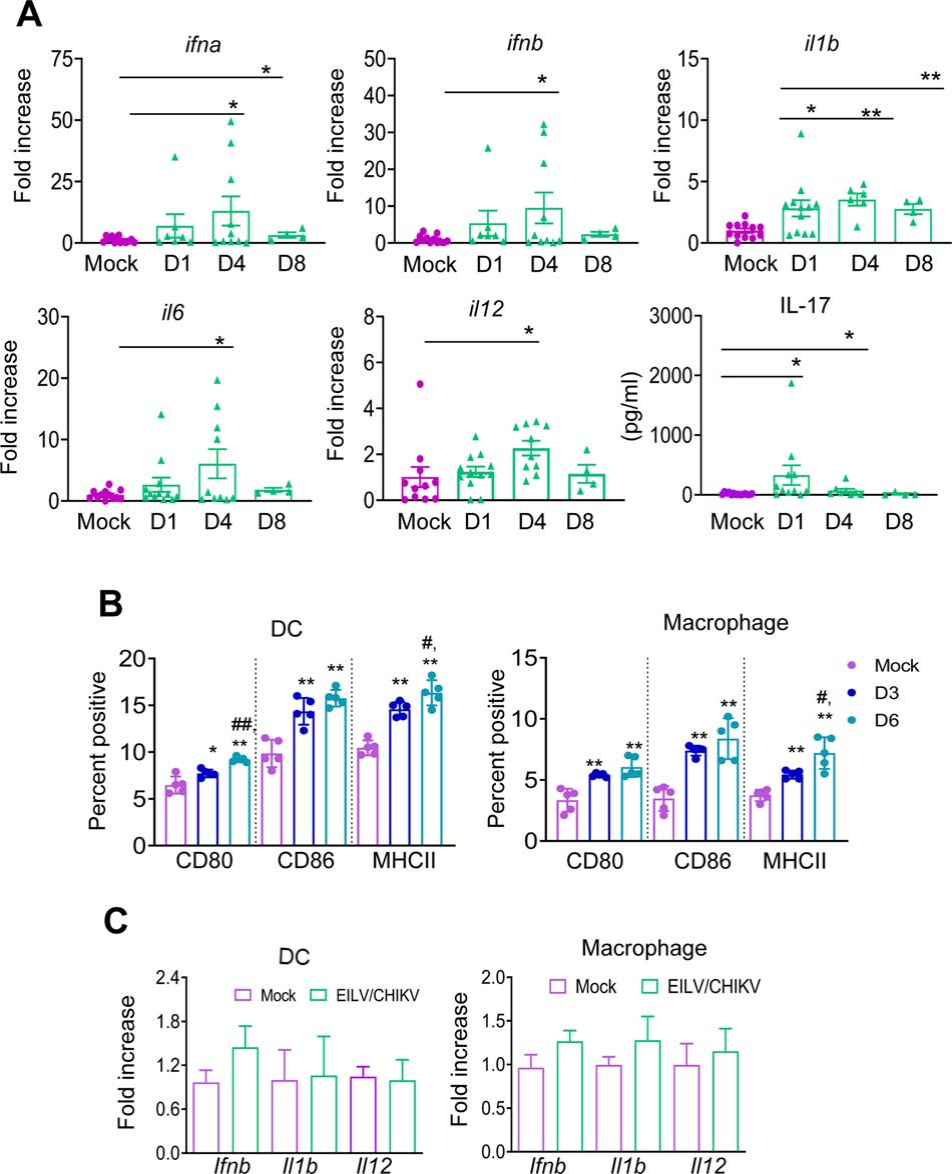
EILV/CHIKV induces potent antiviral cytokine responses and activation of APCs *in vivo,* but does not induce APCs alone upon *in vitro* exposure. 4-week-old B6 mice were infected with 1× 10^8^ PFU EILV/CHIKV or PBS (mock). (A) On days 1, 4 and 8 post-vaccination, blood cytokines levels were determined by Q-PCR assay (*n* = 4–11) or Bioplex assay. Q-PCR results were presented as the fold increase compared to mock- infected. Data are presented as means ± SE and are representative of at least 2 similar experiments. (B) The percentages of CD80^+^ CD11c^+^, CD86^+^CD11c^+^, or MHCII^+^CD11c^+^ for DCs and CD80^+^ F4/80^+^, CD86^+^F4/80^+^, or MHCII^+^F4/80^+^ for macrophages. B6 mice were vaccinated with 10^8^ PFU EILV/CHIKV or PBS (mock). At days 3 (D3) and 6 (D6) post vaccination, splenocytes were stained for cell surface markers, and analyzed by flow cytometry (*n* = 5). Total splenocytes were gated. (C) Bone marrow- derived DCs and macrophages were treated *in vitro* with EILV/CHIKV (MOI = 5). Cells were harvested at day 4 to measure cytokine levels by Q-PCR assay (*n* = 4–10). ** *P* < 0.01 or *P < 0.05 compared to mock group. ^#^*P* < 0.01 or ^##^*P* < 0.01 compared to day 3.

To further understand the underlying mechanisms of immune induction, we characterized the innate immune response to EILV/CHIKV. 4-to- 6-week-old B6 mice were vaccinated with 10^8^ PFU of EILV/CHIKV or PBS. The levels of antiviral innate cytokines, including IFNα, IFNβ, IL-1β, IL-6, and IL-12 in the blood were increased 1.6-to-8-fold in the vaccinated group compared to those of the mock group at day 4 and/or day 8 (Figure 6(A)). Other inflammatory cytokines, such as IL-1β and IL-17 were also up-regulated 2–19-fold at day 1 (Figure 6(A)). Dendritic cells (DCs) and macrophages are important APCs during viral infection. At days 3 and 6 post-vaccination, splenocytes from vaccinated mice were stained with antibodies for DC (CD11c) and macrophage (CD11b/F4/80) markers together with cell maturation and activation molecules, including CD80, CD86 and MHC class II. Compared to the mock group, the levels of CD80, CD86 and MHCII expression on both DCs and macrophages were increased at both time points with a higher magnitude for MHC class II expression at the latter time point (Figure 6(B)). Interestingly, when DCs and macrophages were exposed *in vitro* to EILV/CHIKV (MOI of 5), neither cell type displayed increased levels of gene expression of antiviral innate cytokines, including *Ifnb*, *Il1b* and *Il12* (Figure 6(C)). Thus, it appears that EILV/CHIKV induces anti-viral cytokine responses and APC maturation *in vivo*, but it does not activate APCs upon *in vitro* exposure.

## Discussion

Our prior work showed that EILV/CHIKV, which encodes the replication machinery of EILV and structural proteins of CHIKV, does not replicate in vertebrate cells, but induces robust adaptive immunity and protects animals from WT CHIKV challenge [20]. Here, we further optimized the replication of EILV/CHIKV in C7/10 cells and developed a two-step protocol to isolate highly purified chimeric virus. We also demonstrated that a single dose of 10^8^ PFU of the purified EILV/CHIKV was required to induce strong CHIKV- specific antibody production, MBC, and long-lasting CD8^+^ T cell responses.

Despite multiple platforms being utilized in CHIKV vaccine development, safety and high manufacturing cost have been major concerns [32]. Here, we reported a two-step purification method, starting with chromatography on Cellufine sulfate columns, which facilitates a 100-fold virus concentration and likely its primary purification, followed by ultracentrifugation in discontinuous sucrose gradient. This purification procedure was fast, and highly reproducible. Mass spectrometry also demonstrated the high purity of the chimeric virus stocks with minimal contamination of mosquito-specific proteins. Hypersensitivity reactions were reported in some human volunteers vaccinated with non-purified viral vaccine prepared from C6/36 mosquito culture [33]. To exclude this possibility, we performed a mouse sensitization study and found that the purified EILV/CHIKV did not induce Th1 or Th2 cytokines or more than minimal inflammation in the inoculation sites. In comparison, SGE challenge triggered substantial infiltration of eosinophils and mononuclear cells and T helper cytokine production in the sensitized mice. Thus, the two-step purification procedure of viral stocks prepared from C7/10 cell culture has increased the safety of our candidate vaccine by removal of mosquito proteins.

Both B and T cell-mediated immune responses contribute to host protection against CHIKV infection. RAG1^-/-^ mice, which are deficient of mature B and T cells, develop persistent CHIKV infection [34], suggesting a role of adaptive immunity in viral clearance. Adoptive transfer of CHIKV immune sera into the RAG1^-/-^ mice only transiently reduces infectious virus levels [35]. Furthermore, viral evasion of antiviral CD8^+^ T cell immunity contributes to a persistent CHIKV infection in joint-associated tissue [36], which together suggest that T cells, particularly CD8^+^ T subsets are important for viral clearance during CHIKV infection. Indeed, vaccination with CHIKV-specific CD8^+^ T cell antigens protected mice from footpad swelling and reduced inflammation following wild-type CHIKV infection via the footpad [37]. In contrast, CHIKV-specific CD4^+^ T cells play a pathogenic role by induction of joint swelling without any effect on control of virus replication and dissemination [3,38]. In addition, one group reported that CD4^+^ T cells are nonessential for host protection following vaccination [37]. Here, we found that EILV/CHIKV induces higher CD8^+^ T cell responses than CHIKV181/25 and CHIKV/IRES. EILV/CHIKV-vaccinated mice had lower CD4^+^ T cell responses compared to CHIKV/IRES. Thus, compared to other vaccine candidates, EILV/CHIKV displays stronger immunogenicity but a reduced risk of induction of bystander reactions.

EILV/CHIKV induced the activation of APCs, such as DCs and macrophages, *in vivo*. Interestingly, neither DCs nor macrophages were activated upon *in vitro* exposure to EILV/CHIKV alone, which indicates that other host cell types are involved in early immune induction and facilitate the maturation of APCs in mice. We also noted increased serum IL-17 production in vaccinated mice as early as day 1. The major source for IL-17 during the early stages of infection are γδ T cells [39,40], which rapidly expand, and become activated upon microbial infection [41,42]. The crosstalk between γδ T cells and APCs has been shown to form a unique link between innate and adaptive immune responses [43-46]. Importantly, γδ T cells were also reactive following wild-type CHIKV infection [47]. Thus, future investigation will be focused on the role of γδ T cell activation in promoting APC maturation, and priming CHIKV-specific adaptive immunity upon EILV/CHIKV vaccination. The underlying mechanisms of EILV/CHIKV mediated immune induction remain unclear. During viral infection, cells of the innate immune system utilize pathogen recognition receptor (PRR)s to identify viral pathogens by engaging pathogen-associated molecular patterns and become activated. Although it does not replicate in mammalian cells, EILV/CHIKV was shown to bind and enter Vero cells through the normal endocytic pathway [20]. We speculate that PRRs, such as TLR7 or 8, which are located on the endosome membrane and can detect virus that enter cells after endocytosis, are independent of viral replication, and more likely to be involved in immune induction pathways.

In addition to CHIKV, EILV-based chimeras with other alphaviruses, including eastern and Venezuelan equine encephalitis viruses, also retain their host range restriction in vertebrates but generate strong immune responses [48]. Taken together, the accumulated results demonstrate that the EILV is a safe, inexpensive and effective platform in the development of candidate vaccines for CHIKV and other alphaviruses. Results from this study will also provide novel insights into the mechanism of immune induction by other EILV-based chimeric vaccines. Continued investigation into identifying the intracellular signalling pathways and host factors involved in EILV/CHIKV induction is key for further optimization and development of this novel vector based CHIKV vaccine.

## Supplementary Material

Supplementary_Table_1.xls

## References

[1] Thiberville SD, Moyen N, Dupuis-Maguiraga L, et al. Chikungunya fever: epidemiology, clinical syndrome, pathogenesis and therapy. Antiviral Res. 2013 Sep;99(3):345–370. 23811281 10.1016/j.antiviral.2013.06.009 PMC7114207

[2] Fox JM, Diamond MS. Immune-mediated protection and pathogenesis of Chikungunya virus. J Immunol. 2016 Dec 1;197(11):4210–4218. 27864552 10.4049/jimmunol.1601426 PMC5120763

[3] Poh CM, Chan YH, Ng LFP. Role of T cells in Chikungunya virus infection and utilizing their potential in anti-viral immunity. Front Immunol. 2020;11:287. 32153590 10.3389/fimmu.2020.00287 PMC7046835

[4] Schwameis M, Buchtele N, Wadowski PP, et al. Chikungunya vaccines in development. Hum Vaccin Immunother. 2016 Mar 3;12(3):716–731. 26554522 10.1080/21645515.2015.1101197 PMC4964651

[5] Akahata W, Yang ZY, Andersen H, et al. A virus-like particle vaccine for epidemic Chikungunya virus protects nonhuman primates against infection. Nat Med. 2010 Mar;16(3):334–338. 20111039 10.1038/nm.2105 PMC2834826

[6] Chang LJ, Dowd KA, Mendoza FH, et al. Safety and tolerability of chikungunya virus-like particle vaccine in healthy adults: a phase 1 dose-escalation trial. Lancet. 2014 Dec 6;384(9959):2046–2052. 25132507 10.1016/S0140-6736(14)61185-5

[7] Ramsauer K, Schwameis M, Firbas C, et al. Immunogenicity, safety, and tolerability of a recombinant measles-virus-based chikungunya vaccine: a randomised, double-blind, placebo-controlled, active-comparator, first-in-man trial. Lancet Infect Dis. 2015 May;15(5):519–527. 25739878 10.1016/S1473-3099(15)70043-5

[8] Brandler S, Ruffie C, Combredet C, et al. A recombinant measles vaccine expressing chikungunya virus-like particles is strongly immunogenic and protects mice from lethal challenge with chikungunya virus [Research support. Non-U.S. Gov't]. Vaccine. 2013 Aug 12;31(36):3718–3725. 23742993 10.1016/j.vaccine.2013.05.086

[9] Wressnigg N, Hochreiter R, Zoihsl O, et al. Single-shot live-attenuated chikungunya vaccine in healthy adults: a phase 1, randomised controlled trial. Lancet Infect Dis. 2020 Oct;20(10):1193–1203. 32497524 10.1016/S1473-3099(20)30238-3

[10] Gorchakov R, Wang E, Leal G, et al. Attenuation of Chikungunya virus vaccine strain 181/clone 25 is determined by two amino acid substitutions in the E2 envelope glycoprotein. J Virol. 2012 Jun;86(11):6084–6096. 22457519 10.1128/JVI.06449-11 PMC3372191

[11] Weiss CM, Liu H, Riemersma KK, et al. Engineering a fidelity-variant live-attenuated vaccine for chikungunya virus. NPJ Vaccines. 2020;5:97. 33083032 10.1038/s41541-020-00241-z PMC7560698

[12] Meshram CD, Lukash T, Phillips AT, et al. Lack of nsP2-specific nuclear functions attenuates chikungunya virus replication both in vitro and in vivo. Virology. 2019 Aug;534:14–24. 31163352 10.1016/j.virol.2019.05.016 PMC7204530

[13] Chu H, Das SC, Fuchs JF, et al. Deciphering the protective role of adaptive immunity to CHIKV/IRES a novel candidate vaccine against Chikungunya in the A129 mouse model. Vaccine. 2013 Jul 18;31(33):3353–3360. 23727003 10.1016/j.vaccine.2013.05.059 PMC3731778

[14] Partidos CD, Paykel J, Weger J, et al. Cross-protective immunity against o'nyong-nyong virus afforded by a novel recombinant chikungunya vaccine. Vaccine. 2012 Jun 29;30(31):4638–4643. 22583812 10.1016/j.vaccine.2012.04.099 PMC3372665

[15] Wang E, Kim DY, Weaver SC, et al. Chimeric Chikungunya viruses are nonpathogenic in highly sensitive mouse models but efficiently induce a protective immune response. J Virol. 2011 Sep;85(17):9249–9252. 21697494 10.1128/JVI.00844-11 PMC3165793

[16] Kim DY, Atasheva S, Foy NJ, et al. Design of chimeric alphaviruses with a programmed, attenuated, cell type-restricted phenotype. J Virol. 2011 May;85(9):4363–4376. 21345954 10.1128/JVI.00065-11 PMC3126257

[17] Wang E, Volkova E, Adams AP, et al. Chimeric alphavirus vaccine candidates for chikungunya. Vaccine. 2008 Sep 15;26(39):5030–5039. 18692107 10.1016/j.vaccine.2008.07.054 PMC2571998

[18] Nasar F, Palacios G, Gorchakov RV, et al. Eilat virus, a unique alphavirus with host range restricted to insects by RNA replication. Proc Natl Acad Sci USA. 2012 Sep 4;109(36):14622–14627. 22908261 10.1073/pnas.1204787109 PMC3437828

[19] Nasar F, Gorchakov RV, Tesh RB, et al. Eilat virus host range restriction is present at multiple levels of the virus life cycle. J Virol. 2015 Jan 15;89(2):1404–1418. 25392227 10.1128/JVI.01856-14 PMC4300653

[20] Erasmus JH, Auguste AJ, Kaelber JT, et al. A chikungunya fever vaccine utilizing an insect-specific virus platform. Nat Med. 2017 Feb;23(2):192–199. 27991917 10.1038/nm.4253 PMC5296253

[21] Beaty BJ, Calisher CH, Shope RE. Arboviruses. In: Lennete ET, Lennete DA, editors. Diagnostic procedures for viral, rickettsial and chlamydial infections. 7th ed. Washington, DC: American Public Health Association. 1995. p. 189–212.

[22] Wang T, Town T, Alexopoulou L, et al. Toll-like receptor 3 mediates West Nile virus entry into the brain causing lethal encephalitis. Nat Med. 2004 Dec;10(12):1366–1373. 15558055 10.1038/nm1140

[23] Xie G, Luo H, Pang L, et al. Dysregulation of Toll-like receptor 7 compromises innate and adaptive T cell responses and host resistance to an attenuated West Nile virus infection in old mice. J Virol. 2016 Feb 1;90(3):1333–1344. 26581984 10.1128/JVI.02488-15 PMC4719598

[24] Welte T, Aronson J, Gong B, et al. Vgamma4+ T cells regulate host immune response to West Nile virus infection. FEMS Immunol Med Microbiol. 2011 Nov;63(2):183–192. 22077221 10.1111/j.1574-695X.2011.00840.x PMC3605001

[25] Hallengard D, Lum FM, Kummerer BM, et al. Prime-boost immunization strategies against Chikungunya virus. J Virol. 2014 Nov;88(22):13333–13343. 25210177 10.1128/JVI.01926-14 PMC4249109

[26] Muthumani K, Lankaraman KM, Laddy DJ, et al. Immunogenicity of novel consensus-based DNA vaccines against Chikungunya virus. Vaccine. 2008 Sep 19;26(40):5128–5134. 18471943 10.1016/j.vaccine.2008.03.060 PMC2582145

[27] Erasmus JH, Needham J, Raychaudhuri S, et al. Utilization of an Eilat virus-based chimera for serological detection of Chikungunya infection. PLoS Negl Trop Dis. 2015;9(10):e0004119. 26492074 10.1371/journal.pntd.0004119 PMC4619601

[28] Adam A, Woda M, Kounlavouth S, et al. Multiplexed FluoroSpot for the analysis of Dengue virus- and Zika virus-specific and cross-reactive memory B cells. J Immunol. 2018 Dec 15;201(12):3804–3814. 30413671 10.4049/jimmunol.1800892 PMC6289764

[29] Nasar F, Erasmus JH, Haddow AD, et al. Eilat virus induces both homologous and heterologous interference. Virology. 2015 Oct;484:51–58. 26068885 10.1016/j.virol.2015.05.009 PMC4567418

[30] Schuchman R, Kilianski A, Piper A, et al. Comparative characterization of the Sindbis virus proteome from mammalian and invertebrate hosts identifies nsP2 as a component of the virion and sorting Nexin 5 as a significant host factor for alphavirus replication. J Virol. 2018 Jul 15;92(14):e00694–18. 29743363 10.1128/JVI.00694-18 PMC6026752

[31] Hogrefe WR. Biomarkers and assessment of vaccine responses. Biomarkers. 2005 Nov;10(Suppl 1):S50–S57. 16298912 10.1080/13547500500216629

[32] Schwameis M, Roppenser B, Firbas C, et al. Safety, tolerability, and immunogenicity of a recombinant toxic shock syndrome toxin (rTSST)-1 variant vaccine: a randomised, double-blind, adjuvant-controlled, dose escalation first-in-man trial. Lancet Infect Dis. 2016 Sep;16(9):1036–1044. 27296693 10.1016/S1473-3099(16)30115-3

[33] Scott RM, Shelton AL, Eckels KH, et al. Human hypersensitivity to a sham vaccine prepared from mosquito-cell culture fluids. J Allergy Clin Immunol. 1984 Dec;74(6):808–811. 6150050 10.1016/0091-6749(84)90183-0

[34] Seymour RL, Adams AP, Leal G, et al. A Rodent model of Chikungunya virus infection in RAG1 -/- mice, with features of persistence, for vaccine safety evaluation. PLoS Negl Trop Dis. 2015 Jun;9(6):e0003800. 26115459 10.1371/journal.pntd.0003800 PMC4482609

[35] Hawman DW, Stoermer KA, Montgomery SA, et al. Chronic joint disease caused by persistent Chikungunya virus infection is controlled by the adaptive immune response. J Virol. 2013 Dec;87(24):13878–13888. 24131709 10.1128/JVI.02666-13 PMC3838294

[36] Davenport BJ, Bullock C, McCarthy MK, et al. Chikungunya virus evades antiviral CD8(+) T cell responses to establish persistent infection in joint-associated tissues. J Virol. 2020 Apr 16;94(9):e02036–19. 32102875 10.1128/JVI.02036-19 PMC7163133

[37] Broeckel RM, Haese N, Ando T, et al. Vaccine-induced skewing of T cell responses protects against Chikungunya virus disease. Front Immunol. 2019;10:2563. 31736977 10.3389/fimmu.2019.02563 PMC6834551

[38] Teo TH, Lum FM, Claser C, et al. A pathogenic role for CD4+ T cells during Chikungunya virus infection in mice. J Immunol. 2013 Jan 1;190(1):259–269. 23209328 10.4049/jimmunol.1202177

[39] Chien YH, Meyer C, Bonneville M. Gammadelta T cells: first line of defense and beyond. Annu Rev Immunol. 2014;32:121–155. 24387714 10.1146/annurev-immunol-032713-120216

[40] Roark CL, Simonian PL, Fontenot AP, et al. Gammadelta T cells: an important source of IL-17. Curr Opin Immunol. 2008 Jun;20(3):353–357. 18439808 10.1016/j.coi.2008.03.006 PMC2601685

[41] Ferrick DA, King DP, Jackson KA, et al. Intraepithelial gamma delta T lymphocytes: sentinel cells at mucosal barriers. Springer Semin Immunopathol. 2000;22(3):283–296. 11116958 10.1007/s002810000047

[42] O'Brien RL, Roark CL, Born WK. IL-17-producing gammadelta T cells. Eur J Immunol. 2009 Mar;39(3):662–666. 19283718 10.1002/eji.200839120 PMC2698711

[43] Collins C, Wolfe J, Roessner K, et al. Lyme arthritis synovial gammadelta T cells instruct dendritic cells via fas ligand. J Immunol. 2005 Nov 1;175(9):5656–5665. 16237055 10.4049/jimmunol.175.9.5656

[44] Ismaili J, Olislagers V, Poupot R, et al. Human gamma delta T cells induce dendritic cell maturation. Clin Immunol. 2002 Jun;103(3 Pt 1):296–302. 12173304 10.1006/clim.2002.5218

[45] Leslie DS, Vincent MS, Spada FM, et al. CD1-mediated gamma/delta T cell maturation of dendritic cells. J Exp Med. 2002 Dec 16;196(12):1575–1584. 12486100 10.1084/jem.20021515 PMC2196072

[46] Munz C, Steinman RM, Fujii S. Dendritic cell maturation by innate lymphocytes: coordinated stimulation of innate and adaptive immunity. J Exp Med. 2005 Jul 18;202(2):203–207. 16027234 10.1084/jem.20050810 PMC2213015

[47] Long KM, Ferris MT, Whitmore AC, et al. Gammadelta T cells play a protective role in Chikungunya virus-induced disease. J Virol. 2016 Jan 1;90(1):433–443. 26491151 10.1128/JVI.02159-15 PMC4702549

[48] Erasmus JH, Seymour RL, Kaelber JT, et al. Novel insect-specific Eilat virus-based chimeric vaccine candidates provide durable, mono- and multivalent, single-dose protection against Lethal alphavirus challenge. J Virol. 2018 Feb 15;92(4):e01274–17. 29187545 10.1128/JVI.01274-17 PMC5790933

